# Detection of *Klebsiella pneumoniae* human gut carriage: a comparison of culture, qPCR, and whole metagenomic sequencing methods

**DOI:** 10.1080/19490976.2022.2118500

**Published:** 2022-08-31

**Authors:** Kenneth Lindstedt, Dorota Buczek, Torunn Pedersen, Erik Hjerde, Niclas Raffelsberger, Yutaka Suzuki, Sylvain Brisse, Kathryn Holt, Ørjan Samuelsen, Arnfinn Sundsfjord

**Affiliations:** a Department of Medical Biology, Faculty of Health Sciences, UiT the Arctic University of Norway, Tromsø, Norway; b Norwegian National Advisory Unit on Detection of Antimicrobial Resistance, Department of Microbiology and Infection Control, University Hospital of North Norway, Tromsø, Norway; c Department of Chemistry, UiT the Arctic University of Norway, Tromsø, Norway; d Department of Microbiology and Infection Control, University Hospital of North Norway, Tromsø, Norway; e Department of Computational Biology and Medical Sciences, The University of Tokyo, Tokyo, Japan; f Biodiversity and Epidemiology of Bacterial Pathogens Unit, Institut Pasteur, Université Paris Cité, Paris, France; g Department of Infectious Diseases, Central Clinical School, Monash University, Melbourne, Australia; h Department of Infection Biology, London School of Hygiene and Tropical Medicine, London, UK; i Department of Pharmacy, Faculty of Health Sciences, UiT the Arctic University of Norway, Tromsø, Norway

**Keywords:** Klebsiella pneumoniae, qPCR, whole metagenomic sequencing, gastrointestinal carriage, detection, quantification, sequence type

## Abstract

*Klebsiella pneumoniae* is an important opportunistic healthcare-associated pathogen and major contributor to the global spread of antimicrobial resistance. Gastrointestinal colonization with *K. pneumoniae* is a major predisposing risk factor for infection and forms an important hub for the dispersal of resistance. Current culture-based detection methods are time consuming, give limited intra-sample abundance and strain diversity information, and have uncertain sensitivity. Here we investigated the presence and abundance of *K. pneumoniae* at the species and strain level within fecal samples from 103 community-based adults by qPCR and whole metagenomic sequencing (WMS) compared to culture-based detection. qPCR demonstrated the highest sensitivity, detecting *K. pneumoniae* in 61.2% and 75.8% of direct-fecal and culture-enriched sweep samples, respectively, including 52/52 culture-positive samples. WMS displayed lower sensitivity, detecting *K. pneumoniae* in 71.2% of culture-positive fecal samples at a 0.01% abundance cutoff, and was inclined to false positives in proportion to the relative abundance of other Enterobacterales present. qPCR accurately quantified *K. pneumoniae* to 16 genome copies/reaction while WMS could estimate relative abundance to at least 0.01%. Quantification by both methods correlated strongly with each other (Spearman’s rho = 0.91). WMS also supported accurate intra-sample *K. pneumoniae* sequence type (ST)-level diversity detection from fecal microbiomes to 0.1% relative abundance, agreeing with the culture-based detected ST in 16/19 samples. Our results show that qPCR and WMS are sensitive and reliable tools for detection, quantification, and strain analysis of *K. pneumoniae* from fecal samples with potential to support infection control and enhance insights in *K. pneumoniae* gastrointestinal ecology.

## Plain Language Summary

**What is the context?**

*Klebsiella pneumoniae* is a major cause of healthcare-associated infections and a key contributor to the spread of resistance to last-line antimicrobials.

Gastrointestinal colonization by *K. pneumoniae* is a risk factor for developing infection and can facilitate the spread of antimicrobial resistance.

Culture-based detection may lack sensitivity and is ill-suited to detecting within-sample *K. pneumoniae* abundance and diversity.

Developing molecular methods to improve *K. pneumoniae* abundance and strain diversity detection are essential in understanding human gut colonization and ecology.

**What is new?**

- We compared culture-based detection of *K. pneumoniae* within human fecal samples to two molecular-based techniques: 1) qPCR, which amplifies *K. pneumoniae* species complex-specific DNA targets with high sensitivity, and 2) whole metagenomic sequencing (WMS), which sequences the entire DNA content of complex microbial communities.
- Our findings show:
- qPCR had the highest sensitivity, detecting *K. pneumoniae* in all (52/52) culture-positive samples and 11/51 and 23/47 culture-negative samples, using a direct-fecal and culture-sweep method, respectively. qPCR could accurately quantify *K. pneumoniae* to 16 genome copies/reaction.
- WMS had lower sensitivity, positive in 37/52 culture-positive samples, and demonstrated false positives arising from closely related bacterial species. Relative abundance estimates could be performed to 0.01%.
- WMS performed accurate strain-level detection of *K. pneumoniae* to 0.1% relative abundance and could detect within-sample strain diversity.

What is the impact?

qPCR and WMS are valid methods for the detection and characterization of colonizing *K. pneumoniae* with potential to enhance our understanding of the gastrointestinal ecology of this important pathogen.

## Introduction

*Klebsiella pneumoniae* (Kp) is a critical priority pathogen that has become a major contributor in the spread of antimicrobial resistance (AMR) within and between sectors.^1–4^ Multidrug-resistant (MDR) Kp clones have disseminated globally and are a leading cause of opportunistic healthcare-associated infections, with limited treatment options and high morbidity and mortality rates.^5–8^ In parallel, ‘hypervirulent’ (Hv) Kp clones have emerged which are typically non-MDR, however, can cause invasive community-acquired infections in otherwise healthy individuals.^9^

Kp is part of the phylogenetically broader *Klebsiella pneumoniae* species complex (KpSC), consisting of the seven closely related taxa (or phylogroups): *K. pneumoniae sensu strictu* (Kp1), *K. quasipneumoniae* subsp. *quasipneumoniae* (Kp2) and subsp. *similipneumoniae* (Kp4), *K. variicola* subsp. *variicola* (Kp3) and subsp. *tropica* (Kp5), ‘*K. quasivariicola*’ (Kp6), and *K. africana* (Kp7).^8,10–13^ Kp1 (referred to here as Kp) is of most clinical relevance and consists of a highly diverse population structure corresponding to thousands of unique sequence types (STs) as defined by the seven-gene multi-locus sequence typing (MLST) scheme; https://bigsdb.pasteur.fr/klebsiella/.^8,14^

Kp is a common colonizer of the human gastrointestinal tract. The prevalence of gastrointestinal colonization in the community can range from 4% and 6% in the USA and Australia, 40% and 65% in Senegal and Madagascar, and up to 75% and 87% in Taiwan and Malaysia.^15–18^ We recently described a KpSC carriage rate of 16.3% among 2975 adults in a general urban population in Northern Norway using the KpSC selective Simmon’s citrate agar with inositol (SCAI).^19,20^ Gastrointestinal colonization itself is a major risk factor for invasive infection in hospitalized patients and an increased relative abundance corresponds to a higher infection risk.^15,21–23^ The gut is also an important reservoir for the spread of AMR through clonal dissemination and horizontal gene transfer (HGT).^24,25^

Despite the clinical and epidemiological importance of gastrointestinal carriage, significant knowledge gaps regarding the prevalence, abundance, and diversity of Kp in human gut colonization remain. Kp detection is generally performed by culture-based screening of fecal samples or rectal swabs, which is time-consuming and gives limited information regarding abundance and intra-sample strain diversity. Culture-based detection has also been shown to lack sensitivity in detection of Gram-negative pathogens from fecal samples.^26,27^ Molecular methods such as quantitative PCR (qPCR) and shotgun whole metagenomic sequencing (WMS) offer a potential to compensate for these shortcomings. Both qPCR- and metagenomics-based methods have demonstrated equivalent or improved detection sensitivity for pathogenic bacteria and AMR genes in clinical and environmental samples compared to culture.^26–30^

The aim of this study was to evaluate and compare WMS, qPCR, and culture for the detection and quantification of Kp from human fecal samples at both the species and strain level. Using the extensive culture and whole-genome sequencing (WGS) data gathered during our previous culture-based Kp carriage study, we analyzed a representative selection of Kp culture-positive and negative fecal samples by both qPCR and WMS. Results were compared to culture for Kp detection sensitivity and analyzed for Kp relative abundance and intra-sample strain diversity.

## Results

### Efficiency and sensitivity of the ZKIR-qPCR in human fecal samples

We employed the recently developed ZKIR-qPCR for Kp detection in this study due to its high sensitivity and specificity for KpSC detection in environmental and food samples.^30,31^ BLAST analysis of the 78 bp ZKIR-qPCR target sequence revealed high sequence conservation in all 484 KpSC genomes from our previous cross-sectional carriage study, with 98.6% (477/484) having three or less bp mismatches in the forward primer, and a single conserved A to G substitution at the 5’ end of the reverse primer region.^20^ Importantly the 3’ ends of both forward and reverse primer regions were perfectly conserved, except for a single Kp2 isolate with an A to G substitution at the 3’ terminal end of the forward primer (Suppl. Figure 1). Calculated melting temperatures (*T*_M_) of the PCR product of each sequence variant ranged from 78.8 to 79.9°C.

The ZKIR-qPCR had an amplification efficiency > 90% and R^2^ > 0.99 in a linear dynamic range from 250,000 to 3 genome copies per reaction, both in the presence and absence of 25 ng KpSC-negative fecal microbiome DNA, when assessed against representative strains of each of the four human-associated KpSC subspecies (Kp1-4, Suppl. Table 1, Suppl. Figure 2). Each selected KpSC strain had the most prevalent number of forward primer mismatches seen for that subspecies in the BLAST analysis (Kp1 = 1, Kp3 = 3, Kp2 = 2, Kp4 = 2, Suppl. Figure 1). In-line with Poisson distribution, dilutions below three genome copies per reaction only intermittently detected the ZKIR amplicon.^32^

Limit of Detection (LOD) was three genome copies per reaction for all four KpSC subspecies, both in the presence and absence of 25 ng KpSC-negative fecal microbiome DNA (Suppl. Table 2). At 16 genome copies, copy number could be quantified to a coefficient of variation (CV) ≤ 35%, a previously reported Limit of Quantification (LOQ).^33^

### Detection of Kp in human fecal samples by the ZKIR-qPCR

To determine the Kp detection sensitivity of the ZKIR-qPCR in human fecal samples, 52 Kp culture-positive and 51 KpSC culture-negative human fecal samples were selected from our previous study.^20^ DNA was prepared as a direct fecal microbiome extraction (Direct samples), as well as from a plate-sweep of each sample re-grown on SCAI media (Sweep samples). Four culture-negative samples failed to grow on SCAI media. A total of 61.2% (63/103) Direct and 75.8% (75/99) Sweep samples were positive by the ZKIR-qPCR (Table 1). All culture-positive samples (52/52) were positive by the ZKIR-qPCR in both Direct and Sweep sample preparations. Of the ZKIR-qPCR positive Direct samples, 6.4% (4/63) were not detected after Sweep enrichment. Sanger sequencing performed on seven Direct sample amplicons confirmed the correct ZKIR sequence.

**Table 1.** Comparison of KpSC detection by SCAI culture compared to the ZKIR-qPCR in 103 fecal samples using the Direct fecal microbiome (Direct) and SCAI sweep enrichment (Sweep) DNA extraction methods.

	No. (%) of samples positive by ZKIR-qPCR	
Culture result^a^	*Direct*	*Sweep*
positive n = 52	52 (100)	52 (100)
negative n = 51	11 (22)	23 (49)^b^

Direct, Direct fecal microbiome DNA extraction; Sweep, DNA extraction from a plate sweep of fecal samples cultured 48 hours on SCAI media.

^a^SCAI culture detection result as per our previous culture-based Kp gut carriage study^20^

^b^Four culture negative samples failed to grow on SCAI media, thus % Sweep positive was calculated using n = 47 culture negative samples.

**Figure 1.**
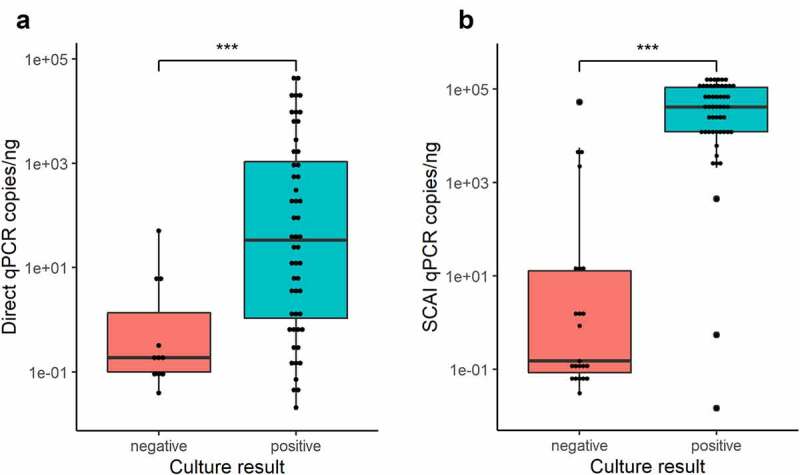
Comparison of KpSC abundance between culture-positive (teal) and culture-negative (red) samples detected by the ZKIR-qPCR using the Direct fecal method (a) and SCAI sweep enrichment (b). *** = *p* < .001 (Mann Whitney U test).

Quantification of genome copy number in Direct samples demonstrated that culture-positive samples had a significantly higher KpSC abundance than culture-negative samples (median 33.72 and 0.17 genome copies/ng DNA, respectively, *p* < .001), (Figure 1a, Suppl. Table 3). This difference was amplified by Sweep enrichment (culture-positive median: 40,865 genome copies/ng DNA, culture-negative median: 0.15 genome copies/ng DNA, *p* < .001), (Figure 1b, Suppl. Table 3).

### Profiling WMS samples and effect of sample collection kit

Taxonomic profiling of whole metagenomic sequenced Direct samples demonstrated the presence of the major human gut associated phyla (Suppl. Table 4A).^34,35^ Enterobacterales, which dominated the Proteobacteria phyla, had a mean of relative abundance of 17.2% (Suppl. Table 4B). This was considerably higher than previously reported mean of ≤ 2% relative abundance for Enterobacterales within gut microbiomes from healthy adults.^34,35^ As expected, Enterobacterales abundance increased to almost complete domination of metagenomes following SCAI sweep enrichment, (mean 95.6%, SD 9%) (Supplementary Table 4C).

To investigate whether our sampling method had caused an artificially increased Enterobacterales abundance, we performed repeat sampling, WMS, and taxonomy profiling of ten participants from our previous culture-based Kp carriage study using the original collection method (ESwabs) and compared these to a validated preservative microbiome sample collection kit (Norgen) (Suppl. Table 5A-D).^36^ Taxonomic comparison revealed significant expansion of Enterobacterales in ESwab samples compared to Norgen samples, (ESwab median 38.2% vs Norgen median 0.62%, *p* = .002, Suppl. Table 5C and D). This was predominantly driven by *Escherichia coli* overgrowth, (median 26.2% vs 0.16% in ESwabs and Norgen samples, respectively, *p* = .002), which constituted a median of 86.8% of total Enterobacterales in ESwabs, compared to 39.8% in Norgen samples. While Kp abundance also underwent a significant increase in ESwabs compared to Norgen samples (median 0.42% vs 0.03%, respectively, *p* = .006), its total relative abundance within Enterobacterales reduced (median 0.99% vs 4.2% in ESwabs and Norgen samples, respectively). The biased microbiome profile caused by collection in ESwabs prevented any Kp-microbiome association analyses to be included as part of this study.

### Detection of Kp by WMS

**Figure 2.**
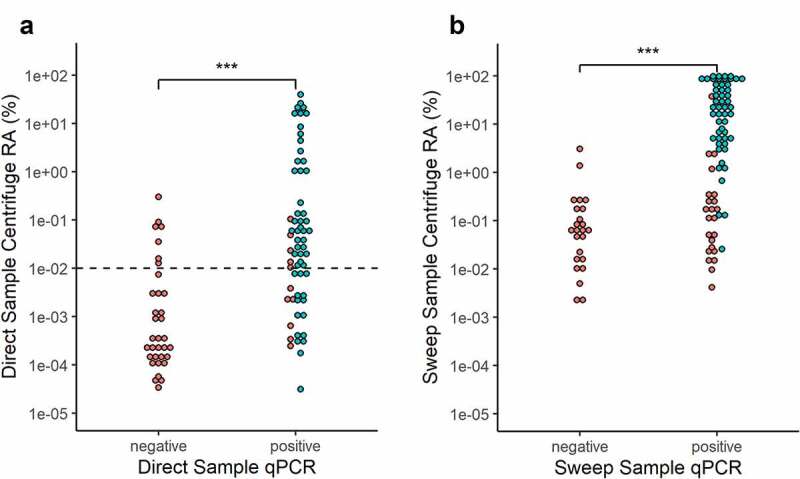
Kp abundance detected by Centrifuge compared to ZKIR-qPCR and SCAI culture detection in (a) Direct and (b) Sweep samples. Teal = SCAI culture positive, Red = SCAI culture negative. RA = relative abundance. Dotted-line line in Figure 2a represents 0.01% abundance detection cutoff used in Direct samples. *** = *p* < .001 (Mann Whitney U test).

To determine the sensitivity and specificity of Kp detection by WMS, samples were analyzed using the taxonomic profiler Centrifuge and compared to the ZKIR-qPCR and culture. Reads were assigned to Kp in all except four Direct samples (n = 99) and all Sweep samples that had grown (n = 99). Two additional Direct samples had < 10^–5^% Kp relative abundance, so were considered as negative. Kp relative abundance in qPCR positive samples (median 0.027% and 6.05% in Direct and Sweep samples, respectively), was significantly higher than qPCR negative samples, (median 0.00035% and 0.063% for Direct and Sweep samples, respectively, both had *p* < .001) (Figure 2, Suppl. Table 3). Despite this, considerable overlap in abundances was observed between qPCR- and culture-positive and negative groups, precluding easy distinction of Kp presence or absence by WMS from either the Direct or Sweep preparations.

We hypothesized many of the qPCR and culture-negative samples with high Kp relative abundance by WMS were false positives misassigned from closely related Enterobacterales species. To investigate this further, two non-Kp containing *in silico* binary species mixes were constructed and Kp abundance measured. Each species mix consisted of reads from *Bacteroides fragilis* and either *E. coli* or *Klebsiella aerogenes* in increasing proportions from 0.1% to 100% relative abundance. An increasing rate of Kp false positives were observed as the abundance of both Enterobacterales species increased, with a higher effect observed for *K. aerogenes* (Suppl. Figure 3), which is the species most closely related to the KpSC. Kp false positives exceeded 0.01% between 10–15% and 30–35% *K. aerogenes* and *E. coli* relative abundance, respectively. At 100% *K. aerogenes* or *E. coli* relative abundance, Kp false positives were 0.20% and 0.056%, respectively.

*E. coli* was the most abundant Enterobacterales species in our Direct samples, with mean relative abundance of 12.1% (Suppl. Table 3). Since Kp false positives over 0.01% did not to appear in our *in silico* species mix until *E. coli* relative abundance was greater than 30%, 0.01% relative abundance was used as a cutoff to report Kp presence to optimize detection sensitivity and specificity. Using this cutoff, Kp was detected in 66.7% (42/63) and 71.2% (37/52) of ZKIR-qPCR and culture-positive Direct samples, respectively (Figure 2a, Suppl. Table 3). Of the 49 Direct samples with Kp WMS abundance above 0.01%, 85.7% (42/49) and 75.5% (37/49) were positive by the ZKIR-qPCR and SCAI culture, respectively. Of the seven Direct samples negative by both qPCR and culture with a Centrifuge Kp abundance above 0.01%, six had Enterobacterales abundances above 15%, suggestive of false positives. Due to the high Enterobacterales abundance in Sweep samples (mean 95.6%), which would be expected to generate high Kp false positives, no such detection cutoff was applied (Suppl. Table 3).

### Screening assembled metagenomes for Kp-specific sequences

**Figure 3.**
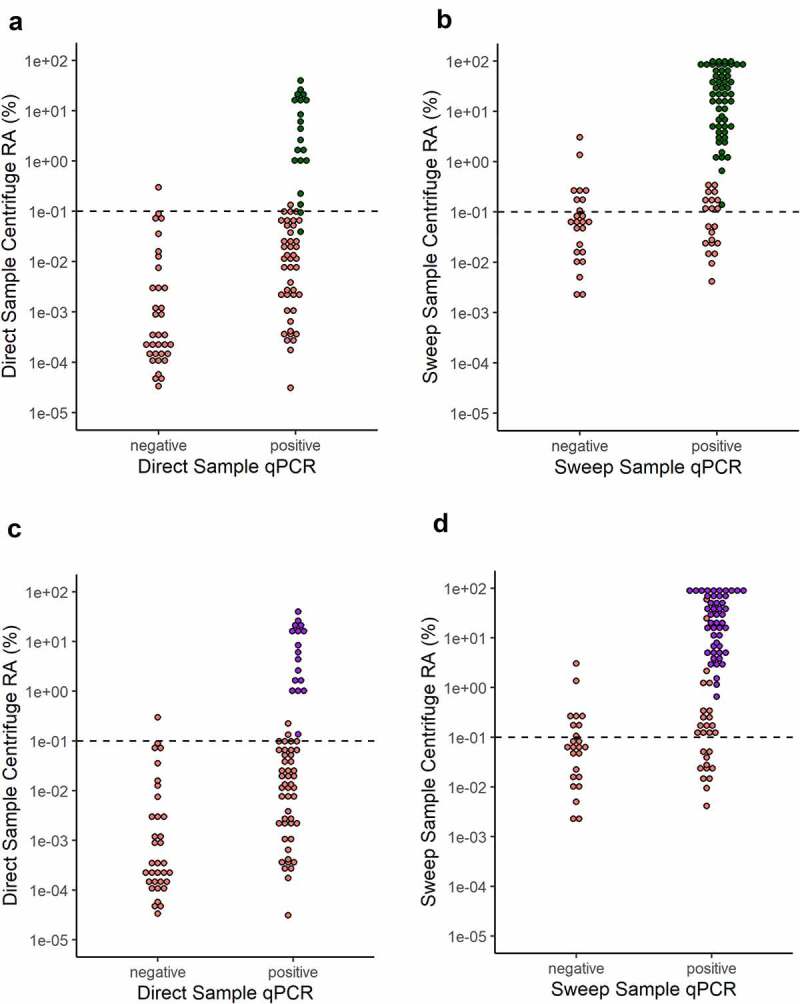
Kp detection from assembled metagenomes using KpSC specific 78bp ZKIR sequence (green) and 4/7 Kp MLST genes (purple) in Direct and Sweep samples. (a) and (b): ZKIR sequence detected in Direct and Sweep samples, respectively. (c) and (d): 4/7 Kp MLST genes detected in Direct and Sweep samples, respectively. Red = ZKIR/MLST sequences not detected. RA = relative abundance. Dotted line at 0.1% Centrifuge abundance represents approximate threshold for detection by these methods.

To use a more specific approach for WMS-based detection, assembled metagenomes were screened for the seven Kp MLST genes and the ZKIR sequence. The ZKIR sequence was detected in 54.5% (54/99) of Sweep samples, all of which were positive by qPCR and 92.6% (50/54) were positive by culture (Figure 3b, Suppl. Table 3). Similarly, using a detection cutoff of 4/7 MLST genes, 52.5% (52/99) Sweep samples were positive, all of which were also positive by qPCR and 94.2% (49/52) were positive by culture (Figure 3d, Suppl. Table 3). Detection sensitivity was considerably lower in Direct samples, with 19.4% (20/103) and 16.5% (17/103) positive by ZKIR sequence and 4/7 MLST gene detection, respectively. All Direct ZKIR and MLST positive samples, however, were positive by both qPCR and culture (Figure 3 a and c, Suppl. Table 3). All Sweep and Direct samples with at least 4/7 MLST genes detected were also positive for the ZKIR sequence. Detection by these methods had clear dependence on Kp abundance, with the ZKIR sequence detected in only two samples below approximately 400 genome copies/ng DNA by qPCR and 0.1% relative abundance by WMS, while 4/7 MLST genes were not detected in any samples below this threshold.

### Kp relative abundance estimation by WMS and correlation with qPCR

**Figure 4.**
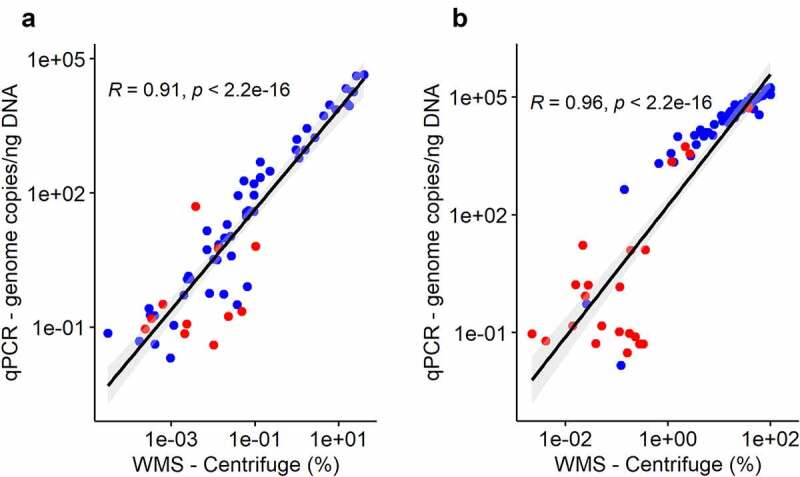
Correlation of Kp abundances quantified by qPCR vs WMS (Centrifuge) in Direct samples (a) and Sweep samples (b). R = Spearman’s rho. Blue = SCAI culture positive, Red = SCAI culture negative.

KpSC abundance measured by qPCR had a strong correlation with Kp relative abundance measured by Centrifuge in both ZKIR-qPCR positive Direct and Sweep samples (Spearman’s rho = 0.91, and 0.96 respectively, both with p < .001), (Figure 4). Sweep samples also demonstrated clear separation between culture-positive and negative samples by both qPCR and Centrifuge (Figure 4b). In these samples, below a limit of approximately 2000 copies/ng by qPCR and 0.6% by Centrifuge, only 9.5% (2/21) of samples were detected as positive by culture, compared to 92.5% (50/54) above this threshold.

The accuracy of Kp abundance quantification by WMS was investigated using two *in vitro* Kp-spiked microbiomes that consisted of: 1) a mock microbiome of six bacterial species representing the major gut taxa, including 1% *E. coli* to represent total Enterobacterales, and 2) a KpSC-negative human fecal microbiome with 0.52% total Enterobacterales abundance. Both microbiomes were spiked with Kp at 1%, 0.1%, 0.01%, and 0.001%, and 0% relative abundance. The mock microbiome was spiked with a single strain (ST11), while the fecal microbiome was spiked with a combination of three Kp strains (ST11, ST23, and ST101), at a ratio of 60:30:10, respectively (Suppl. Table 1).

Centrifuge achieved close estimations of all Kp relative abundances, differing from spiked abundances by a factor of 0.46 to 0.86 and 3.52 to 4.79 in the mock and fecal microbiomes, respectively (Table 2 and Table 3). Background Kp abundances measured in the non-spiked mock and fecal microbiomes were 0.0004% and 0.00068%, respectively, which were close to the observed Kp false positives in the *in silico* binary species mixes containing 0.5% and 1% total Enterobacterales abundances (Suppl. Figure 3). These findings suggest in ‘healthy’ microbiomes, which typically contain <1% total Enterobacterales abundance, Kp quantification can be performed to as low as 0.01% without substantial influence from false positives.^34,35^

**Table 2.** *K. pneumoniae* abundance in the spiked mock microbiome measured by WMS (Centrifuge) and the ZKIR-qPCR.

Sample	Kp spike(%)	WMS measured abundance (%)	Ratio spiked/measured	ZKIR-qPCR ave copies/reaction
M1	0.00	0.0004	NA	0
M2	0.001	0.00086	0.86	41
M3	0.01	0.0046	0.46	412
M4	0.1	0.050	0.50	4776
M5	1.0	0.59	0.59	61395

**Table 3.** *K. pneumoniae* abundance in the spiked fecal microbiome measured by WMS (Centrifuge) and the ZKIR-qPCR.

Sample	Kp spike (%)	WMS measured abundance (%)	Ratio spiked/measured	ZKIR-qPCR ave copies/reaction
F1	0.00	0.00068	NA	0
F2	0.001	0.0035	3.52	16
F3	0.01	0.038	3.78	235
F4	0.1	0.43	4.34	2905
F5	1.0	4.79	4.79	31925

NA = not applicable

The average genome copy numbers measured by the ZKIR-qPCR in the 0.001% spiked mock and fecal microbiomes of 41 and 16 genome copies/reaction, respectively, were close to the measured LOQ for this assay. This suggests at 0.001% relative abundance the ZKIR-qPCR is approaching the lower limit at which it can accurately quantify Kp abundance.

### Kp strain-level detection from metagenomic data

Metagenomic Kp strain-level analysis of Direct samples was performed with StrainGST, part of the StrainGE toolkit.^37^ To examine the tool’s accuracy, we explored our Kp-spiked mock and fecal microbiomes described above (Table 4 and Table 5). The spiked Kp isolate ST11 was correctly identified in the mock microbiome at 1% and 0.1% abundance. In the fecal microbiome, the three spiked isolates, ST11, ST23, and ST101, were all correctly identified at 1%, while ST11 and ST23 only were identified at 0.1%. No strains were detected in either spiked microbiome at abundances below 0.1%, in line with the reported lower detection limit for this tool.^37^ A spiked mock microbiome sample containing 1% Kp (ST11) and 1% Kp3 (ST697) was also tested, in which both strains were identified correctly. Estimated abundances of each spiked strain by StrainGST were close approximations of the true abundances. No false positives were reported in the spiked fecal microbiome samples. In the Kp spiked mock microbiomes ST12 and ST340 were reported in the samples spiked with 1% and 0.1% ST11, respectively. Comparison of the MLST profiles revealed ST12 and ST340 are single and double locus variants of ST11, respectively.

**Table 4.** Metagenomic Kp strain-level detection performed using StrainGST in a Kp-spiked mock microbiome sample.

Spiked Microbiome			StrainGST result			
Mock	Spiked strain(s)	Spiked abundance (%)	Strain(s) detected	Strain est. abundance (%)	Total est. abundance (%)	Confidence Score
M1	none	NA	ND	0	0	NA
M2	ST11	0.001	ND	0	0	NA
M3	ST11	0.01	ND	0	0	NA
M4	ST11	0.1	ST12^a^, ST11	0.051, 0.166	0.217	0.207, 0.044
M5	ST11	1.0	ST11, ST340^a^	0.429, 0.762	1.191	0.760, 0.027
M6	ST11, ST697^b^	1:1	ST11, ST697, ST340^a^	0.798, 0.377, 0.756	1.93	0.705, 0.591, 0.03

^a^ST340 and ST12 are single and double locus variants of ST11, respectively

^b^*Klebsiella variicola*

NA = not applicable

ND = not detected.

**Table 5.** Metagenomic Kp strain-level detection performed using StrainGST in a Kp-spiked fecal microbiome sample.

Spiked Microbiome			StrainGST result			
Fecal	Spiked strains (ratio 60:30:10)	Spiked abundance (%)	Strain(s) detected	Strain est. abundance (%)	Total est. abundance (%)	Confidence Score
F1	none	NA	ND	0	0	NA
F2	ST11, ST23, ST101	0.001	ND	0	0	NA
F3	ST11, ST23, ST101	0.01	ND	0	0	NA
F4	ST11, ST23, ST101	0.1	ST11, ST23	0.056, 0.028	0.084	0.245, 0.029
F5	ST11, ST23, ST101	1.0	ST11, ST23, ST101	0.398, 0.171, 0.2	0.769	0.747, 0.230, 0.082

NA = not applicable

Since no spiked Kp strains were detected below 0.1%, all Direct samples with Centrifuge Kp relative abundance ≥ 0.1%, including two Kp culture-negative samples, were selected for StrainGST analysis, (n = 21 samples, median Kp relative abundance: 2.64%, range: 0.1% – 39.55%, Suppl. Table 3). Kp strains were detected in all culture-positive samples (n = 19), and matched culture detected strains in 84.2% (16/19) (Table 6). Kp strains detected which did not match culture were either four or five locus variants of their culture detected counterparts, suggesting these were not closely related. No strains were detected in either of the two culture-negative samples. Both of these samples had high Enterobacterales abundance (59.6% and 63.9%), while one was qPCR negative and the other had only seven genome copies/ng DNA detected, suggestive of falsely high relative abundance estimated by Centrifuge. Multiple strains were reported in four samples, three with two strains and one had three strains. Three of these samples had strains matching culture-detection, which were the highest confidence and abundance strain in each case. The three strains detected in sample 45 and the two strains in sample 75 were double and triple locus variants of each other, respectively, and considered possibly to be one strain that had been assigned to multiple reference genomes in the database. The two strains detected in both samples 46 and 100 shared only a single MLST locus each, likely representing true microbiome Kp strain diversity.

**Table 6.** Metagenomic Kp strain-level detection performed using StrainGST in 21 adult fecal samples with Kp relative abundance ≥ 0.1%.

T7 Sample	SCAI culture result^a^		Kp Abundance		StrainGST result		
Number	Kp detected?	Strain	WMS (%)^b^	qPCR (copies/ng DNA)	Strain(s) detected	est. abundance (%)	Confidence Score
89	yes	**ST14**	39.55	45900	**ST14**	30.639	0.94
101	yes	ST2042	26.04	39390	ST2039	27.521	0.94
75	yes	**ST485**	22.09	18240	**ST485**, ST35^c^	6.146, 3.875	0.86, 0.66
92	yes	**ST35**	20.55	18660	**ST35**	12.651	0.96
74	yes	**ST27**	17.81	9120	**ST27**	4.061	0.98
18	yes	**ST4039**	15.29	9940	**ST4039**	5.27	0.85
91	yes	**ST1496**	14.73	21240	**ST1496**	14.655	0.98
45	yes	**ST25**	8.39	7400	**ST25**, ST2549, ST4039^c^	0.37, 2.75, 1.16	0.76, 0.1, 0.02
97	yes	**ST704**	6.02	9410	**ST704**	7.38	0.74
12	yes	**ST70**	4.37	5290	**ST70**	6.922	0.65
44	yes	**ST23**	2.64	1716	**ST23**	1.524	0.67
100	yes	ST375	1.73	2785	ST2042, ST1660	2.07, 0.6	0.63, 0.03
46	yes	**ST25**	1.56	920	**ST25**, ST461	0.29, 0.09	0.58, 0.16
2	yes	**ST3043**	1.12	604	**ST3043**	0.32	0.84
90	yes	**ST1106**	0.98	1612	**ST1106**	1.84	0.28
84	yes	**ST200**	0.95	926	**ST200**	0.98	0.67
80	no	NA	0.30	0	ND	NA	NA
72	yes	**ST20**	0.22	298	**ST20**	0.59	0.18
21	yes	**ST151**	0.13	492	**ST151**	1.85	0.22
35	yes	ST25	0.13	217	ST10	0.13	0.33
62	no	NA	0.10	7	ND	NA	NA

Samples in bold represent ST matches between culture detection and strainGST detection

^a^SCAI culture detection result as per our previous culture-based Kp gut carriage study^20^

^b^WMS relative abundance measured by Centrifuge

^c^STs detected in these samples were double (sample 45) and triple (sample 75) locus variants of each other which may have resulted from assignment a single strain to multiple closely related reference genomes in the database rather than true intra-sample strain diversity.

NA = not applicable

NA = not applicable

## Discussion

The purpose of this study was to investigate WMS and the ZKIR-qPCR as methods for the detection and analysis of Kp from human fecal samples and compare these to culture-based detection. Overall, the ZKIR-qPCR demonstrated the highest Kp detection sensitivity which was reflected in the very low LOD of this assay of just three genome copies per reaction. This corresponds to the lowest possible limit for qPCR according to Poisson distribution, indicating the very high efficiency of this assay.^32^ The fact that no culture-positive samples were negative by the ZKIR-qPCR indicates a low false negative rate. Our findings suggest SCAI-based detection may underestimate the true prevalence of KpSC gastrointestinal carriage. This may be related to technical challenges identifying KpSC given other common Enterobacterales, including *Enterobacter, Citrobacter, Serratia*, and other non-KpSC *Klebsiella* species, are capable of growth on SCAI media often with similar morphologies to KpSC.^19,38,39^ In-line with our findings, the ZKIR-qPCR has previously demonstrated a higher KpSC detection sensitivity compared to SCAI culture in plant, soil, chicken meat, and salad samples.^30,31^ Similar to the findings by Barbier *et al*.^30^ we found a culture enrichment step prior to qPCR significantly enhanced detection sensitivity. Together these results demonstrate the ZKIR-qPCR is a rapid and highly accurate tool for KpSC detection in a range of sample types, thereby facilitating targeted selection of samples for further culture- or metagenomics-based analysis.

It is noteworthy that the high detection sensitivity of the ZKIR-qPCR was achieved despite our tested KpSC strains having one (Kp1), two (Kp2 and Kp4), and three (Kp3) bp mismatches within 15 nucleotides of the 5’ end of the forward ZKIR primer and one mismatch at the penultimate position of the 5’ end of the reverse primer. In accordance with these findings, it has been shown up to five bp mismatches within primers can be well tolerated provided the 3’ primer region is well conserved.^40–42^

Accurate species-level profiling is essential for high quality shotgun WMS analyses. Using the taxonomic profiling tool Centrifuge with a 0.01% relative abundance cutoff, Kp was detected at the species level in 66.7% and 71.2% of qPCR and culture-positive Direct samples, respectively. Despite the comparatively high reported sensitivity and specificity of Centrifuge, however, like other metagenomic classifiers it is known to generate false positives at lower species abundances.^43–45^ Using *in silico* binary species mixes, we demonstrated the rate of Kp false positives is proportional to the abundance and relatedness of other Enterobaterales species present in the sample, surpassing 0.01% Kp false positives at 10–15% *K. aerogenes* and 30–35% *E. coli* relative abundance, respectively. Within a ‘healthy’ gut microbiome, in which the average Enterobacterales abundance is ≤2%, this may not significantly impact Kp detection specificity.^34,35^ Much higher Enterobacterales abundance can occur in dysbiotic states including inflammatory bowel disease, type 2 diabetes mellitus, and following antimicrobial therapy.^46–48^ Detection of low abundance Kp by taxonomic classifiers in these settings would therefore need careful interpretation.

Gastrointestinal microbiomes with an increased Kp relative abundance are associated with an increased risk of Kp bacteremia, nosocomial transmission, and may predispose to prolonged colonization.^22,23,49,50^ Accurate measurement of Kp abundance could therefore provide important clinical information relevant for infection risk stratification and infection control purposes. In our Kp spiked microbiomes, which contained ≤1% total Enterobacterales, we found WMS gave close estimations of Kp relative abundance to 0.01%, below which false positives began to have a substantial influence. The ZKIR-qPCR, however, accurately quantified Kp to as low as 16 genome copies/reaction, corresponding to approximately 0.001% relative abundance in the Kp-spiked microbiomes, with the additional advantage of providing quantification information in a clinically relevant timeframe.

The spread of AMR by Kp is predominantly driven by the expansion of MDR high-risk clones disseminating between hospitals and across borders.^1,8,51–55^ The utility of WMS in Kp infection control thus requires timely and sensitive Kp strain-level detection. Using StrainGST, part of the StrainGE toolkit, we demonstrated fast and accurate strain-level detection can be achieved from fecal metagenomes to Kp abundances as low as 0.1%, matching culture-detected strains in 16/19 samples.^37^ Interestingly, only one ST type was detected in most samples, suggesting gut microbiomes may be largely dominated by a single Kp strain. This contrasts with recent small sample sized culture-based studies in which multiple carriage strains were found with much higher frequency.^56–58^ More robust longitudinal studies are needed to determine whether these culture-detected strains represent true gastrointestinal colonizers versus low abundance transitory passengers that are being enriched by culture. Alternatively, the partial enrichment of Enterobacterales, including Kp, as shown in ESwab compared to DNA preserved Norgen samples, may have led to a single ST-type selection overwhelming strain diversity in our Direct samples.

Although we demonstrated high accuracy of Kp strain detection by WMS in our samples, two false positive STs, ST12 and ST340, were detected in our mock microbiome in addition to the spiked strain, both of which were closely related to the spiked ST11. These may have arisen from the stricter database clustering we used to increase resolution between closely related ST types, e.g., ST11 and ST258, resulting in assignment of a single strain to multiple closely related reference genomes. Mismatches between the Kp strain detected by culture and WMS also occurred on three occasions. Whether these differences were the result of misassignment by StrainGE, or alternatively, overgrowth of a low abundance non-dominant strain induced by culture, requires further study.

Our findings suggest a lower limit of 0.1% relative abundance for reliable retrieval of Kp-specific strain and allele-level information from metagenomes, as metagenomic detection of the ZKIR sequence, the Kp MLST alleles, and Kp ST-level detection occurred very seldomly below this level. While this is a considerable level of sensitivity, it nevertheless represented less than half of our Direct samples, which had a median Kp relative abundance of 0.007%, suggesting Kp gastrointestinal carriage typically occurs at lower abundances than this threshold. Our target sequencing depth of 20 million paired-end reads per sample, while shown to be sufficient for species-level detection, may have limited the amount of information recoverable from our samples at the subspecies-level.^59,60^ Performing deeper sequencing in large-scale metagenomic studies, however, is challenging due to high costs and data storage and processing requirements. Strain-level detection performed on culture-enriched samples, such as our SCAI Sweep samples, using tools such as StrainGST or the recently described mSweep/mGEMS pipeline, or through targeted enrichment of metagenomes by RNA-probe hybridization-capture, may provide the most sensitive and cost-effective method for high-resolution strain analysis from metagenomes.^61,62^ Studies are currently underway to explore these important possibilities.

Samples used in this study were initially collected for culture-based purposes, thus ESwab collection devices were used to maintain bacterial viability. The extensive KpSC culture and associated single colony WGS data gathered from these samples made them ideal for the purpose of this study. This collection method was also a major limitation, as it resulted in significant overgrowth of Enterobacterales, particularly *E. coli*, as shown when compared to the validated Norgen collection method. The resultant biased ESwab microbiome meant no Kp-microbiome association analyses could be performed from this data, nor any strong conclusions drawn regarding the normal relative abundance range of Kp gastrointestinal carriage. Further studies are currently underway utilizing the methods described here using validated microbiome collection devices to address these important questions.

In conclusion, we have shown the ZKIR-qPCR and WMS are reliable tools for detection and quantification of Kp within human gastrointestinal samples. Both methods exhibited differing and complementary strengths and weaknesses. This is evidenced by the speed, high sensitivity, and low cost of the ZKIR-qPCR, allowing targeted selection of samples for WMS, which, although less sensitive and more time and resource intensive, can provide in-depth microbiome and strain-level Kp analysis. Future studies using the methods evaluated herein therefore have great potential to enhance our understanding of Kp gastrointestinal ecology. Placed into a One Health context, these approaches will help in elucidating the role of the gastrointestinal tract of humans and animals in the spread of Kp and associated AMR genes between niches.

## Materials and Methods

### Human fecal samples

Fecal samples were drawn from a collection of 2975 KpSC culture-screened samples obtained during our cross-sectional KpSC carriage study and the seventh survey of the Tromsø Study, (The Tromsø Study: Tromsø7).^20^ Briefly, samples were self-collected from community-based adult participants using a nylon-flocked ESwab 490CE.A (Copan, Brescia, Italy). Upon arrival to the laboratory, 200 μL of 85% glycerol was added and samples were stored at −80°C. Samples were screened for KpSC on SCAI media and suspect colonies underwent KpSC identification by MALDI-TOF.^19^ Confirmed KpSC isolates underwent WGS and MLST-typing by Kleborate.^63^ 103 samples were selected for the current study based on i) the presence/absence of Kp as confirmed by WGS, (n = 52 and 51, respectively) and ii) less than 2 days transit time from initial collection to freezing at −80°C. Prior to this study samples had undergone one freeze-thaw cycle.

### Sample preparation and DNA isolation

After thawing on ice, 50 μL of each fecal sample was plated on SCAI media (Sigma-Aldrich, cat # 85462–500 G and I5125-500 G) and incubated at 37°C for 48 hours. Remaining sample was centrifuged (4000 x *g* for 10 minutes at 4°C) and pellet used for a whole microbiome DNA extraction (Direct sample). All growth on SCAI plates was scraped using a 10 μL inoculation loop, and approximately 50 μL (one loaded inoculation loop) used for a SCAI culture sweep DNA extraction (Sweep sample). DNA extractions were performed using the Purelink Microbiome DNA Purification kit (Thermo Fisher Scientific, cat# A29790), according to the manufacturer’s instructions, with the following minor modifications: Step 1a) samples resuspended in 800 μL S1 Lysis Buffer plus 20 mg/mL lysozyme (Thermo Fisher Scientific, cat# 89833) and incubated at 37°C for 10 minutes. Step 1c) following addition of S2 Lysis Enhancer, samples were incubated at 95°C for 10 minutes. Step 1e) samples were homogenized in lysing matrix E tubes (MP Bio, cat# 6914050) using a Precellys Evolution tissue homogenizer (6500 rpm for 2 × 23 s at 4°C) (Bertin Technologies, Montigny Le Bretonneux, France), followed by 2 rounds of centrifugation at 14000 x *g* for 5 min. Step 1 h) prior to addition of S3 Cleanup Buffer, 2 μL of 10 mg/mL RNase A (Thermo Fisher Scientific, cat# EN0531) was added and samples were incubated at room temperature for 5 minutes. Quality control of purified DNA was performed using Nanodrop 2000 spectrophotometer (Thermo Fisher Scientific, Waltham, USA) and concentration determined with Qubit 3.0 fluorometer (Thermo Fisher Scientific).

### In silico analysis of ZKIR target region

BLAST analysis of the 78 bp ZKIR region was performed against the 484 assembled KpSC genomes from our previous carriage study (BioProject: PRJEB42350) using default nucleotide-nucleotide BLAST parameters (NCBI-blast v2.10.0+).^20,64^ Melting temperature (*T*_M_) for each ZKIR variant was calculated using the oligo analysis tool available at: https://eurofinsgenomics.eu/en/ecom/tools/oligo-analysis/. Final amplicon sequences for *T*_M_ calculation consisted of forward and reverse ZKIR primers plus the 30 bp intervening region from each ZKIR sequence variant found by BLAST analysis.

### ZKIR-qPCR assays

#### Reaction mixture, primers, and cycling conditions

PCR mixture, ZKIR primers, and cycling conditions were as described by Barbier *et al*.^30^ All qPCR reactions were performed on an Applied Biosystems 7500 Real-Time PCR System (Thermo Fisher Scientific).

#### Standard curve

ZKIR-qPCR standard curves were prepared using whole genome sequenced representatives of each of the four human-associated KpSC members: Kp1, Kp2, Kp3 and Kp4 (Suppl. Table 1). Strains were grown overnight on tryptose blood agar with lactose/bromothymolblue (Thermo Fisher Scientific cat# CM0233, Thermo Fisher Scientific cat# LP0070, VWR cat# 1.03026.0025) at 37°C, and DNA extraction performed as described. Seven five-fold dilutions of genomic DNA (gDNA) were made for each isolate at 2.5x10^5^, 5x10^4^, 10^4^, 2x10^3^, 400, 80, 16 and 3 genome copies per qPCR reaction, according to the equation: genome copy number = [(mass of input DNA in ng) * (6.0221*10^23^ molecules/mole)]/(length of genome in bp * 660 g/mol * 10^9^ng/g), where length of Kp genome = 5.5x10^6^ bp.^65^ Each dilution point was performed in technical triplicate. Reactions were performed both with and without addition of 25 ng of human fecal microbiome DNA from a healthy donor which was KpSC-negative by the ZKIR-qPCR. Slope, reaction efficiency, R^2^, Y-intercept, and melting temperatures (*T*_M_) were calculated using 7500 Real-Time PCR Analysis Software v2.3 (Applied Biosystems, Life technologies, Waltham, USA).

#### Limit of Detection (LOD) and Limit of Quantification (LOQ)

Limit of Detection (LOD) is defined by the Minimum Information for the Publication of Quantitative Real-Time PCR Experiments as the lowest concentration of target detectable with reasonable certainty.^66^ LOD was therefore taken as the lowest number of genome copies detectable in ten out of ten technical replicates. LOQ was estimated as the lowest dilution at which the coefficient of variation (CV) of genome copy number of ten technical replicates was ≤ 35%, where genome copy number = *[1+(efficiency/100)]^y-^^Cq^*, and CV = [(standard deviation of genome copy number)/(average of genome copy number)] * 100.^33^ LOD and LOQ were performed using gDNA from each of the four KpSC strains above with and without addition of 25 ng KpSC negative human fecal microbiome DNA. ZKIR-qPCR assays were performed as described for Standard Curve, with an additional dilution point at eight genome copies/reaction, and dilution points 16, 8 and 3 genome copies/reaction were performed in 10 technical replicates.

### Detection of Kp by the ZKIR-qPCR

All Direct and Sweep DNA samples diluted to 10 ng/μL and 2.5 μL (25 ng) was used as input for each qPCR reaction. Reaction mixture and cycling conditions were as described previously.^30^ Samples were assayed in technical triplicate and considered positive if amplicons were produced in at least two with a *T*_M_ between 78.3°C and 80.4°C and C_q_ < 40. *T*_M_ range was based on values from the *in silico* analysis of KpSC isolates (described above) ± 0.5°C for inter-assay variability between predicted and measured values. Although microbial detection by qPCR requires amplicons to be present in only a single technical replicate, we increased this threshold to two positive replicates to minimize false positives.^32,33^ Non-template controls were used in all qPCR experiments. Additionally, *E. coli* underwent all processing steps from DNA extraction to ZKIR-qPCR assay in parallel with Direct samples, and *Klebsiella oxytoca* underwent all steps from culture on SCAI media, DNA extraction and ZKIR-qPCR in parallel with Sweep samples (Suppl. Table 1). As both these species do not contain the ZKIR sequence, this controlled for cross-contamination of KpSC DNA between samples at any of the sample processing steps.

### WMS sample processing and analysis

#### Library preparations and sequencing

DNA was fragmented using the Focused-ultrasonicator M220 (Covaris, Woburn, USA). 100ng of fragmented DNA underwent library preparation using TrueSeq Nano DNA Library Prep Kit (Illumina, cat# 20015965) and Swift Turbo 2S flex DNA Library Prep Kit (Swift Biosciences, cat# 45096) in accordance with the manufacturer’s instructions. Sequencing was performed on the NovaSeq 6000 platform (Illumina, San Diego, USA) to a target depth of 20 × 10^6^ pair-end reads at 150 bp.

#### Data processing

FASTQ files underwent removal of optical duplicates using Clumpify (version 38.82), a part of the BBmap package (version 38.79), removal of adapters and poor-quality sequences by fastp (version 0.20.1), and removal of human DNA residues by FastQ Screen (version 0.14.0) against the GRCh38 reference assembly (accession number GCF_000001405.39).^67–69^ Unpaired reads were synchronized by the Repair tool of BBmap package (version 38.79).^67^

#### WMS assembly and Taxonomic profiling

Paired-end and singleton reads were assembled into contigs using MetaSPAdes (v3.13.0) with default parameters.^70^ Kp detection and estimation of abundance was performed using the taxonomy profiler Centrifuge (version 1.0.4) with the default database, p_compressed+h + v.^44^ Centrifuge uses a Burrows-Wheeler transform (BWT) and Ferragina-Manzini (FM) index to create a comparatively small reference database by concatenating and compressing multiple genomes of the same species for rapid and accurate species identification.^44^ For other taxonomic profiling, Kraken 2 (version 2.1.2) and Bracken (version 2.6.1) with the MiniKraken DB_8GB v202003 were used.^71,72^

#### Screening WMS assemblies for Kp-specific sequences

The seven Kp MLST alleles, downloaded from the PasteurMLST database, and the 78bp ZKIR sequence were used as reference databases for identification of Kp in the WMS-assembled contigs.^14^ To screen the contigs, nucleotide BLAST (v2.10.1) was used with DNA identity and coverage parameters set to ≥ 95% (MLST allele detection) or default parameters (ZKIR sequence detection).^64^

#### Computational resources

All computational analyses were performed on the Norwegian academic high-performance computing and storage services maintained by the Sigma2 Norwegian Research Infrastructure Service (NRIS).^73^ Data was stored and shared in the Norwegian e-infrastructure for Life Science (NeLS) maintained by ELIXIR Norway.

### Validation of ESwab versus Norgen collected fecal samples

Ten previous Tromsø7 participants were re-recruited as part of an ongoing longitudinal Kp gut carriage study. Participants were sampled using ESwabs, under the same conditions as the original collection including less than 2 days from sample collection to arrival at the laboratory, and compared to collection taken at the same time using the Norgen Nucleic Acid Preservation system (Norgen Biotek, cat# 53700). All samples underwent library preparation using MGIEasy FS DNA Library Prep Set v2.1 (MGI Tech Co, cat# 1000005254) on the 7-MGISP-960 automated library preparation system (software version: V1.2.0.163, automation version: V1.0), as per manufacturer’s instructions (MGI Tech Co, Shenzen, China). Sequencing was performed on the G400 platform (MGI Tech Co). Processing of sequenced reads and taxonomic profiling was performed as described.

### In silico binary species mixes

FASTQ sequence reads from *B. fragilis, K. aerogenes*, and *E. coli* (Suppl. Table 1) were retrieved from the Sequence Read Archive (NCBI) and subsampled using SEQTK (https://github.com/lh3/seqtk/blob/master/README.md). Subsampled reads were combined to create two binary species mixes containing reads from *B. fragilis* and either *K. aerogenes* or *E. coli* in the following ratios: 99.99/0.01, 99.95/0.05, 99/1, 95/5, 90/10, 75/25, 50/50, 25/75, 0/100. Binary species mixes underwent processing and taxonomy profiling as described samples above.

### Kp-spiked microbiomes

#### Kp-spiked mock microbiome

The mock microbiome was constructed from six bacterial strains: *Bacteroides vulgatus, Clostridium septicum, Bifidobacterium longum, Helicobacter pylori, Aeromonas hydrophila*, and *E. coli* (Suppl. Table 1). gDNA was extracted from each strain and combined in the following relative abundance calculated on genome copy numbers: 40% *B. vulgatus*, 40% *C. septicum*, 10% *B. longum*, 5% *H. pylori*, 4% *A. hydrophila*, and 1% *E. coli*. Abundances represented typical relative abundance of major phyla found in a healthy adult gut microbiome.^34,35^ Kp ST11 gDNA was spiked into six mock microbiome aliquots at relative abundance: 0%, 0.001%, 0.01%, 0.1%, and 1%, as well as 1% Kp ST11 plus 1% Kp3 ST697 (Suppl. Table 1).

#### Kp-spiked fecal microbiome

Whole microbiome DNA was extracted from a fecal sample collected from a healthy adult donor using the Norgen Stool Nucleic Acid Preservation system (Norgen Biotek, cat# 53700) and confirmed KpSC negative by the ZKIR-qPCR. Total bacterial abundance was estimated by qPCR quantification of the bacterial 16S gene using the universal 16S primers described by Clifford *et al*.^65^ qPCR reaction mixture, cycling conditions were as described by Barbier *et al*.^30^ 0.25ng microbiome DNA was used as input, and standard curve was set up as for the ZKIR-qPCR above except the following five-fold dilution series was used: 1.25x10^6^, 2.5x10^5^, 5x10^4^, 10^4^, 2x10^3^, 400, 80, and 16 genome copies per qPCR reaction. gDNA from Kp ST11, ST23, and ST101 (Suppl. Table 1) were combined in the ratio 60:30:10, respectively, and spiked into aliquots of the donor microbiome DNA at 0%, 0.001%, 0.01%, 0.1%, and 1%. All Kp-spiked mock and fecal microbiome samples underwent WMS sequencing, sample processing, and taxonomic analysis as described.

### Kp strain analysis

Kp strain analysis was performed using StrainGST, part of the Strain Genome Explorer (StrainGE) toolkit.^37^ A custom database of KpSC genomes (n = 3604) was constructed with default k-mer size 23. The database consisted of i) all Kp genomes from refseq (NCBI) (n = 1010), downloaded on the 02/02/2022 using NCBI Genome Downloading Scripts, (https://github.com/kblin/ncbi-genome-download), ii) 484 KpSC genomes from our KpSC carriage study (303 Kp1, 134 Kp3, 31 Kp2, and 16 Kp4 genomes), and iii) 2109 KpSC genomes from the recent SPARK study (1705 Kp1, 279 Kp3, 76 Kp2, and 49 Kp4 genomes).^20,74^ The default lower limit for database clustering of 0.90 k-mer similarity resulted in closely related ST types co-clustered (e.g., ST11 and ST258), thus, a lower limit of 0.95 was used for final database clustering.

### Statistical analysis

Statistical differences between sample groups were determined using a one-tailed Mann Whitney U test (independent samples) or one-sided Wilcoxon signed-rank test (paired samples) using Jasp version 0.14.1 (University of Amsterdam, Amsterdam, Netherlands) (https://jasp-stats.org/download/). Correlation analysis of qPCR and Centrifuge Kp abundances was performed using R Studio version 3.6.1. *p*-values <0.05 were regarded as statistically significant.

## Supplementary Material

Supplemental Material

## Data Availability

Metagenomic data (raw Illumina/MGI reads) are publicly available in ENA under BioProject: PRJEB52877.
