# Supplementary material for: Detection of Klebsiella pneumoniae human gut carriage: a comparison of culture, qPCR, and whole metagenomic sequencing methods
Source: Gut Microbes. 2022 Aug 31;14(1):2118500. doi: 10.1080/19490976.2022.2118500 (PMC9450895; doi:10.1080/19490976.2022.2118500)
Supplement: Supplemental Material [file KGMI_A_2118500_SM9138.zip › 20220816 Revised Supplementary material comparative study of Kp detection methods_Gut Microbes.docx]

# Supplementary Material

Detection of *Klebsiella pneumoniae* human gut carriage: a comparison of culture, qPCR, and whole metagenomic sequencing methods

Kenneth Lindstedt (ORCID **0000-0002-5919-2479**)^a*^, Dorota Buczek (ORCID 0000-0002-2282-6298)^a^, Torunn Pedersen (ORCID 0000-0002-9593-8119)^b^, Erik Hjerde (ORCID 0000-0002-6014-1249)^c^, Niclas Raffelsberger (ORCID 0000-0002-9463-8915)^a,d^, Yutaka Suzuki (0000-0003-4852-1879)^e^, Sylvain Brisse (ORCID 0000-0002-2516-2108)^f^, Kathryn Holt (ORCID 0000-0003-3949-2471)^g,h^, Ørjan Samuelsen (ORCID 0000-0002-5525-2614)^b,i^, Arnfinn Sundsfjord (ORCID **0000-0002-3728-2270)**^a,b*^

^a^Department of Medical Biology, Faculty of Health Sciences, UiT The Arctic University of Norway, Tromsø, Norway; ^b^Norwegian National Advisory Unit on Detection of Antimicrobial Resistance, Department of Microbiology and Infection Control, University Hospital of North Norway, Tromsø, Norway; ^c^Department of Chemistry, UiT The Arctic University of Norway, Tromsø, Norway; ^d^Department of Microbiology and Infection Control, University Hospital of North Norway, Tromsø, Norway; ^e^Department of Computational Biology and Medical Sciences, The University of Tokyo, Japan; ^f^Institut Pasteur, Université Paris Cité, Biodiversity and Epidemiology of Bacterial Pathogens, Paris, France; ^g^Department of Infectious Diseases, Central Clinical School, Monash University, Melbourne, Australia; ^h^Department of Infection Biology, London School of Hygiene and Tropical Medicine, London, UK; ^i^Department of Pharmacy, Faculty of Health Sciences, UiT The Arctic University of Norway, Tromsø, Norway

*Corresponding authors: Kenneth Lindstedt, email: [kenneth.w.lindstedt@uit.no](mailto:kenneth.w.lindstedt@uit.no), and Arnfinn Sundsfjord, [arnfinn.sundsfjord@uit.no](mailto:arnfinn.sundsfjord@uit.no). Department of Medical Biology, Faculty of Health Sciences, UiT The Arctic University of Norway, Tromsø, 9038, Norway

# Table of Contents

Supplementary Table 1. Bacterial strains used in this study.

Supplementary Table 2. Comparison of Limit of Detection (LOD) and Limit of Quantification (LOQ) of four human-associated KpSC members (Kp1-4) by the ZKIR-qPCR.

Supplementary Table 3. Comparison of Kp detection by SCAI culture to the ZKIR-qPCR and WMS (provided as Excel file).

Supplementary Table 4. Taxonomic comparison of samples to two large microbiome profiling reference studies (provided as Excel file).

Supplementary Table 5. Taxonomic comparison of Norgen and Eswab samples to two large microbiome profiling reference studies (provided as Excel file).

Supplementary Figure 1: Blast analysis of the 78bp ZKIR-qPCR target sequence against the 484 KpSC isolates detected during our previous Kp population carriage study.

Supplementary Figure 2: ZKIR-qPCR standard curves generated from representative isolates of each of the four human associated KpSC species.

Supplementary Figure 3: Proportion of reads misassigned to the Kp genome by WMS (Centrifuge) from related Enterobacterales in *in silico* binary species mixes with increasing Enterobacterales abundance.

**Supplementary Table 1.** Bacterial strains used in this study

| **Strain** | **Species** | **Use** | **Reference** |
| --- | --- | --- | --- |
| K47-25 (ST258) | Kp1 | ZKIR qPCR reaction efficiency/standard curve | ^1^ |
| T7-004 (ST681) | Kp3 | ZKIR qPCR reaction efficiency | ^2^ |
| T7-071 (ST4653) | Kp2 | ZKIR qPCR reaction efficiency | ^2^ |
| T7-021 (ST4625) | Kp4 | ZKIR qPCR reaction efficiency | ^2^ |
| ATCC 25922 | *E. coli* | ZKIR qPCR negative control (Direct samples) | NA |
| ATCC 43863 | *K. oxytoca* | ZKIR qPCR negative control (Sweep samples) | NA |
| DSM 2151 | *B. fragilis* | *In silico* binary species mix | SRR16258999 |
| DSM 30053 | *K. aerogenes* | *In silico* binary species mix | SRR15076202 |
| ETEC H10407 | *E. coli* | *In silico* binary species mix | ERR2910163 |
| ATCC 8482 | *B. vulgatus* | Mock microbiome | NA |
| ATCC 12464 | *C. septicum* | Mock microbiome | NA |
| UNN S1 | *B. longum* | Mock microbiome | ERS1507214^3^ |
| ATCC 43504 | *H. pylori* | Mock microbiome | NA |
| ATCC 7966 | *A. hydrophila* | Mock microbiome | NA |
| ATCC 35218 | *E. coli* | Mock microbiome | NA |
| K66-45 (ST11) | Kp1 | Kp-spike strain | ^4^ |
| T7-263 (ST697) | Kp3 | Kp3-spike strain | ^2^ |
| T7-442 (ST23) | Kp1 | Kp-spike strain | ^2^ |
| P19-10 (ST101) | Kp1 | Kp spike strain | ^5^ |

**Supplementary Table 2.** Comparison of Limit of Detection (LOD) and Limit of Quantification (LOQ) of four human-associated KpSC members (Kp1-4) by the ZKIR-qPCR.

| **Species** | **Limit of Detection (LOD)^a^** | | **Limit of Quantification (LOQ)^a^** | |
| --- | --- | --- | --- | --- |
|  | gDNA only | gDNA with microbiome DNA^b^ | gDNA only | gDNA with microbiome DNA^b^ |
| *K. pneumoniae* (Kp1) | 3 genomes | 3 genomes | 16 genomes  **CV = 23.5%** | 16 genomes  **CV = 16.7%** |
| *K. variicola* (Kp3) | 3 genomes | 3 genomes | 16 genomes  **CV = 28.2%** | 16 genomes  **CV = 28.51%** |
| *K. quasipneumoniae subsp. quasipneumoniae* (Kp2) | 3 genomes | 3 genomes | 16 genomes  **CV = 35.2%** | 16 genomes  **CV = 27.5%** |
| *K. quasipneumoniae subsp. similipneumoniae* (Kp4) | 3 genomes | 3 genomes | 16 genomes  **CV = 23.5%** | 16 genomes  **CV = 21.78%** |

^a^Limits were defined as lowest genome copy number which could be detected (LOD) or quantified (LOQ) in 10/10 technical replicates.

^b^Assays were performed as isolate genomic DNA (gDNA) only as well as challenged by the presence of 25 ng KpSC negative faecal microbiome DNA.

CV = coefficient of variation.

**Supplementary 3, 4, and 5.** Provided as Excel table


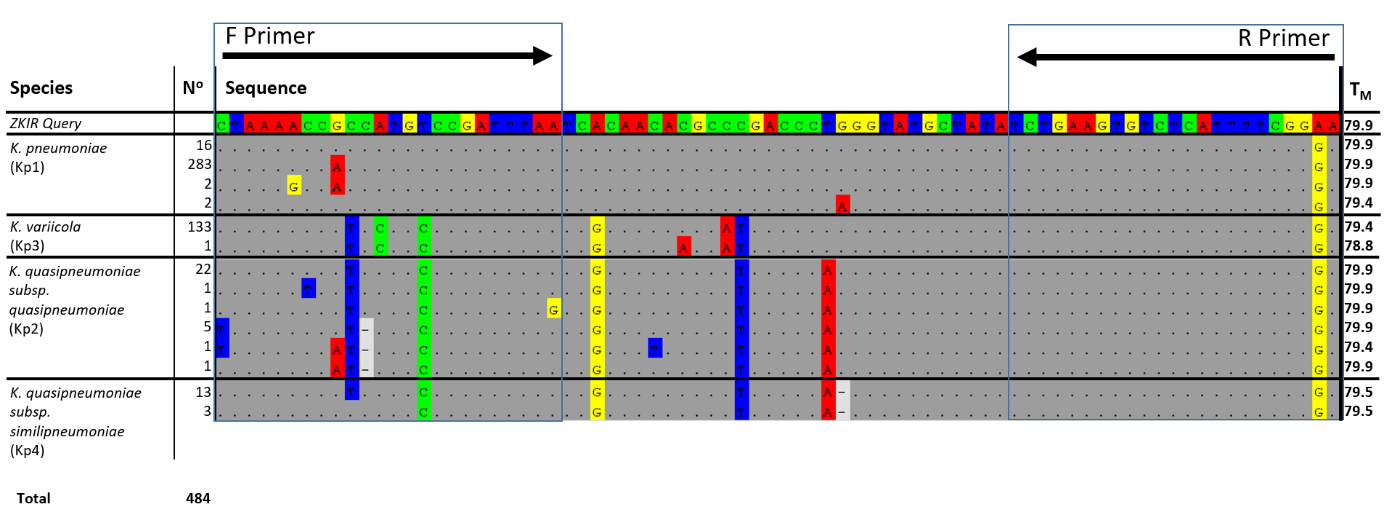


**Supplementary Figure 1:** Blast analysis of the 78bp ZKIR-qPCR target sequence against the 484 KpSC isolates detected during our previous Kp population carriage study.^2^ Base-pair mismatches are highlighted. Calculated amplicon melting temperatures (T_M_) (^o^C) for each sequence variant are shown. Results visualised using AliView Alignment Viewer and Editor (v1.26).

**Supplementary Fig 2:** ZKIR-qPCR standard curves generated from representative isolates of each of the four human associated KpSC species. Assays were performed on isolate gDNA only (A) and in the presence of 25 ng KpSC negative faecal microbiome DNA (B) in technical triplicates from 250,000 genomes/reaction to 3 genomes per reaction. C_q_ = Quantification Cycle


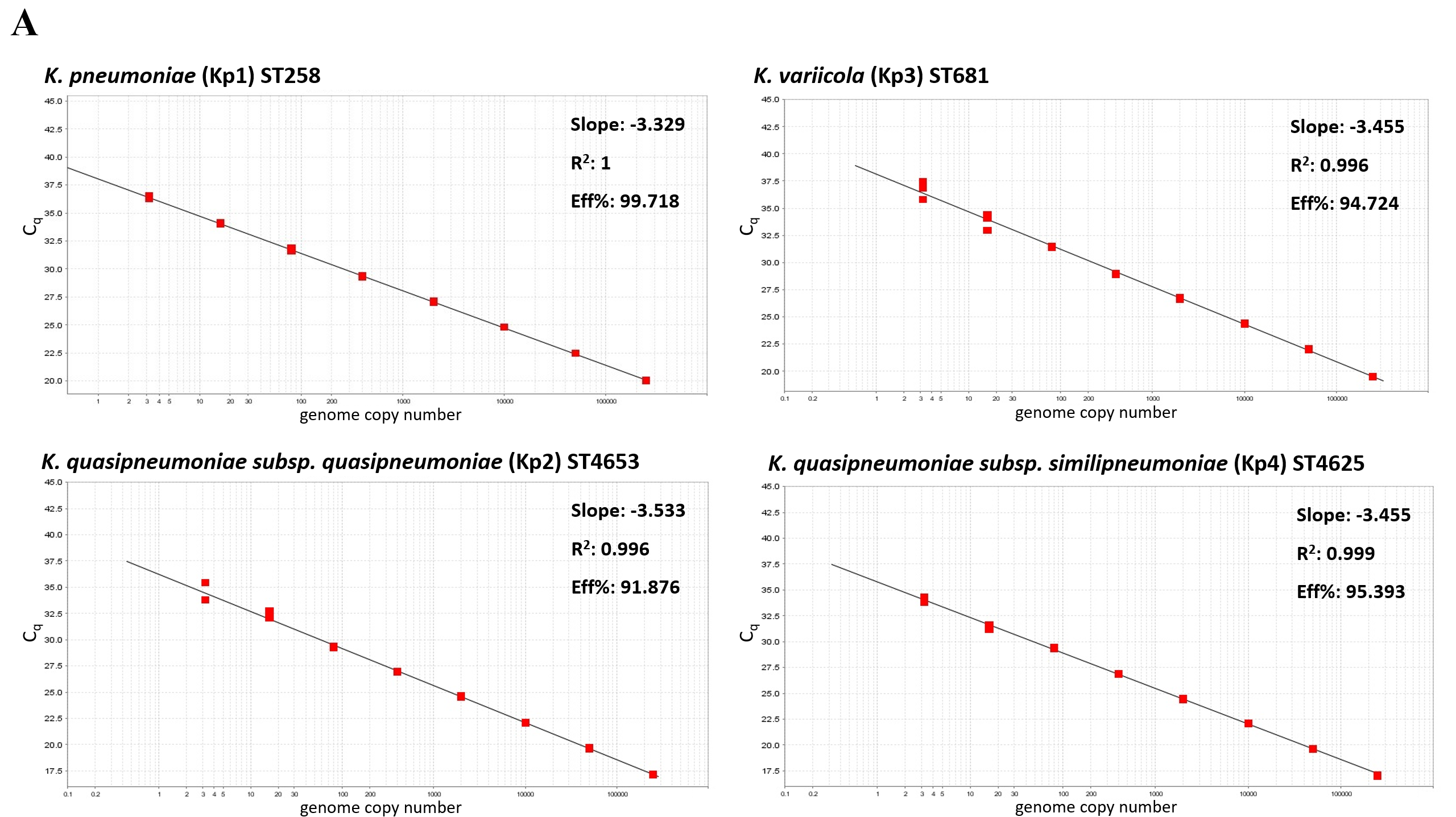

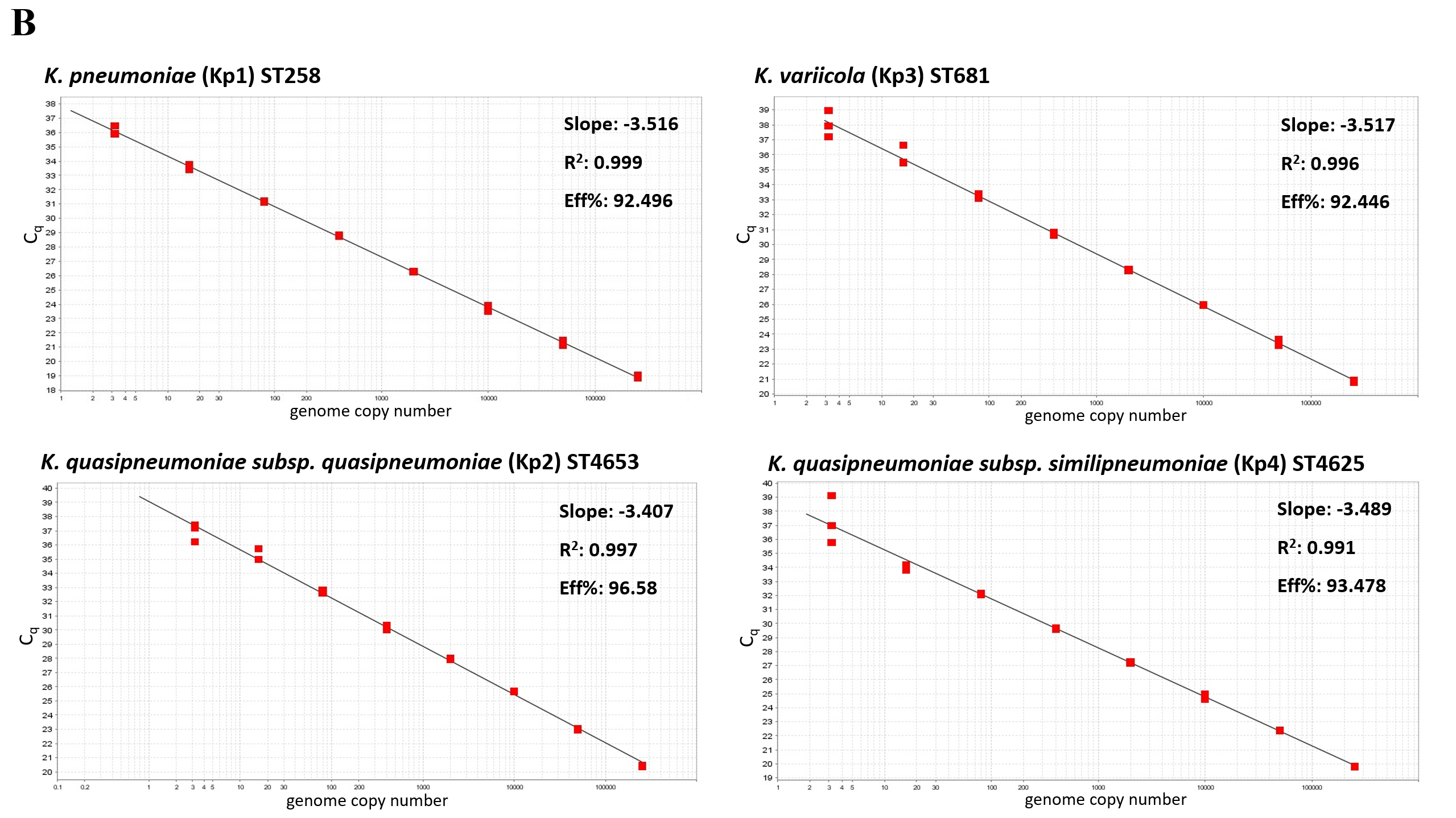


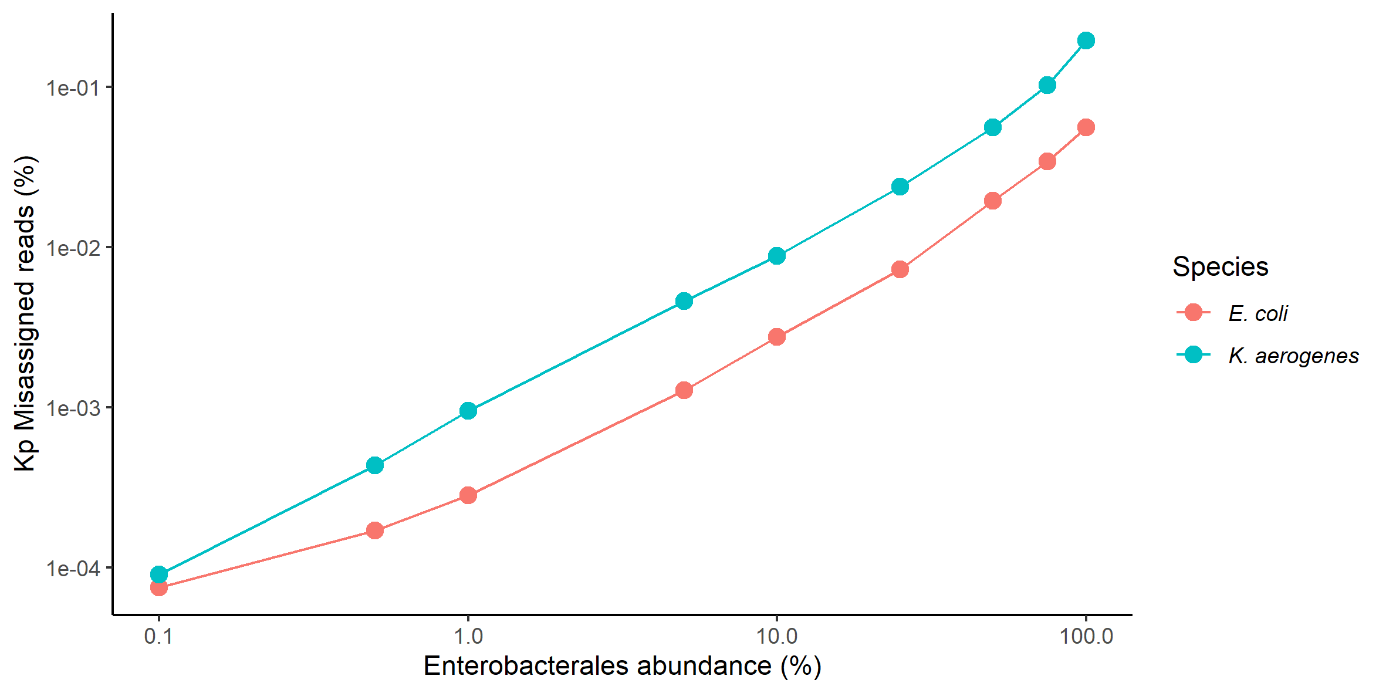
**Supplementary Figure 3:** Proportion of reads misassigned to the *Kp* genome by Centrifuge from related Enterobacterales in *in silico* binary species mixes containing *B. fragilis* and increasing abundance of either *E. coli* (teal) or *K. aerogenes* (orange).

# References

1. Samuelsen O, Naseer U, Tofteland S, Skutlaberg DH, Onken A, Hjetland R, et al. Emergence of clonally related Klebsiella pneumoniae isolates of sequence type 258 producing plasmid-mediated KPC carbapenemase in Norway and Sweden. J Antimicrob Chemother 2009; 63:654-8.

2. Raffelsberger N, Hetland MAK, Svendsen K, Smabrekke L, Lohr IH, Andreassen LLE, et al. Gastrointestinal carriage of Klebsiella pneumoniae in a general adult population: a cross-sectional study of risk factors and bacterial genomic diversity. Gut Microbes 2021; 13:1939599.

3. Esaiassen E, Hjerde E, Cavanagh JP, Simonsen GS, Klingenberg C, Norwegian Study Group on Invasive Bifidobacterial I. Bifidobacterium Bacteremia: Clinical Characteristics and a Genomic Approach To Assess Pathogenicity. J Clin Microbiol 2017; 55:2234-48.

4. Heikal A, Samuelsen O, Kristensen T, Okstad OA. Complete Genome Sequence of a Multidrug-Resistant, blaNDM-1-Expressing Klebsiella pneumoniae K66-45 Clinical Isolate from Norway. Genome Announc 2017; 5.

5. Pedersen T, Sekyere JO, Govinden U, Moodley K, Sivertsen A, Samuelsen O, et al. Spread of Plasmid-Encoded NDM-1 and GES-5 Carbapenemases among Extensively Drug-Resistant and Pandrug-Resistant Clinical Enterobacteriaceae in Durban, South Africa. Antimicrob Agents Chemother 2018; 62.
