# Supplementary figures and images for: Detection of Klebsiella pneumoniae human gut carriage: a comparison of culture, qPCR, and whole metagenomic sequencing methods
Source: Gut Microbes. 2022 Aug 31;14(1):2118500. doi: 10.1080/19490976.2022.2118500 (PMC9450895; doi:10.1080/19490976.2022.2118500)

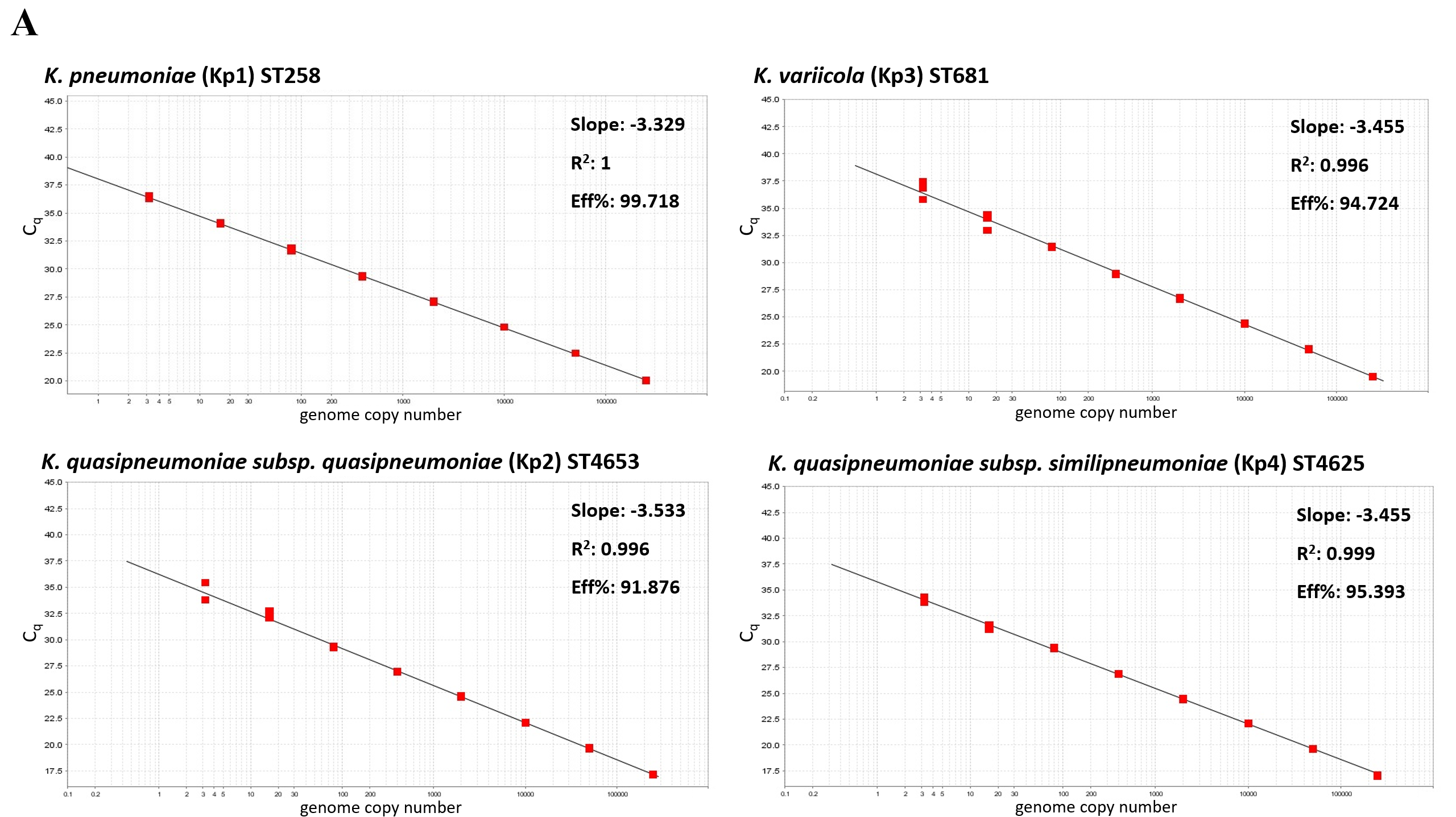

Supplement: Supplemental Material [file KGMI_A_2118500_SM9138.zip › Revised Supplementary Figure 2A.png]

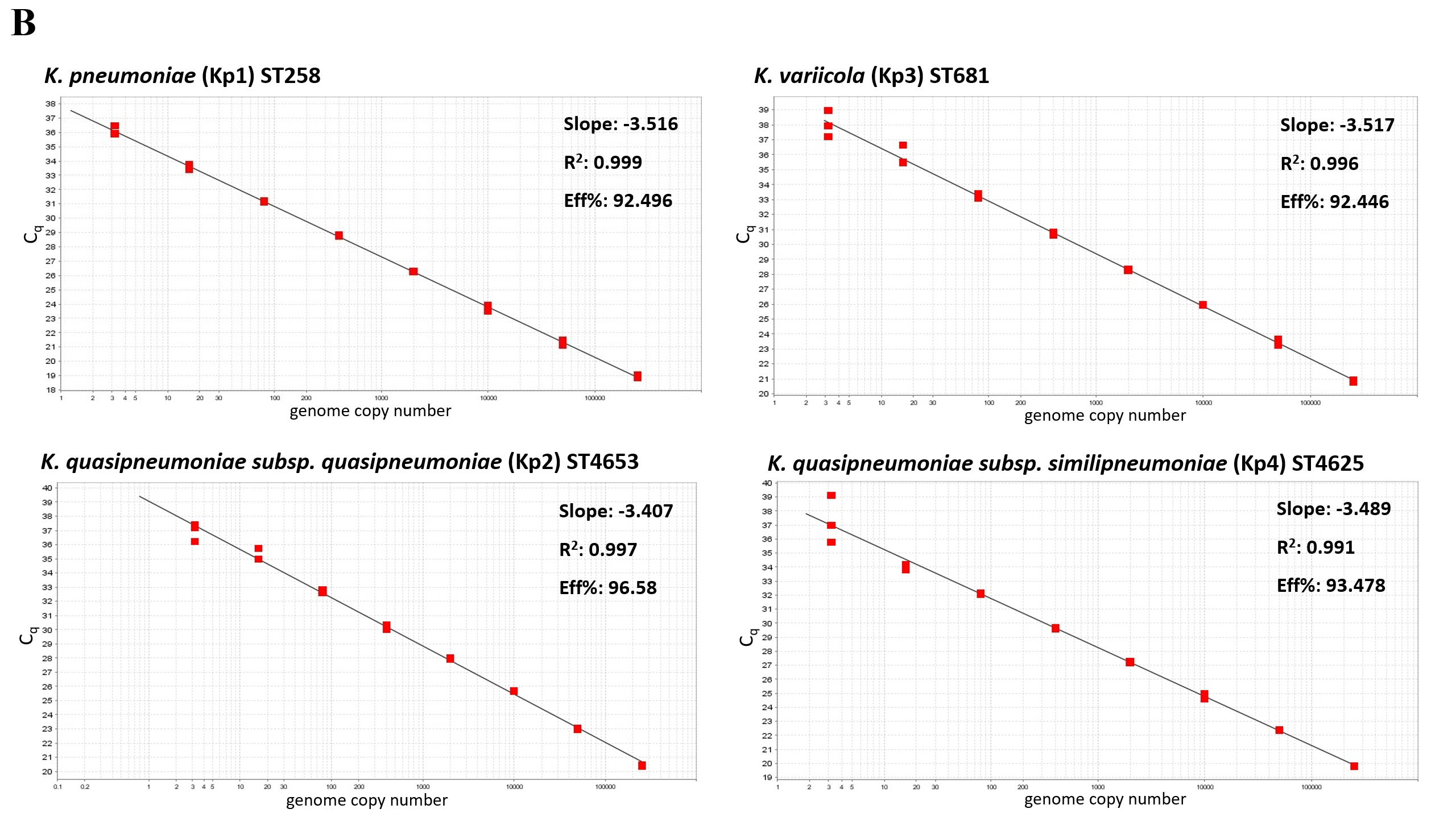

Supplement: Supplemental Material [file KGMI_A_2118500_SM9138.zip › Revised Supplementary Figure 2B.png]

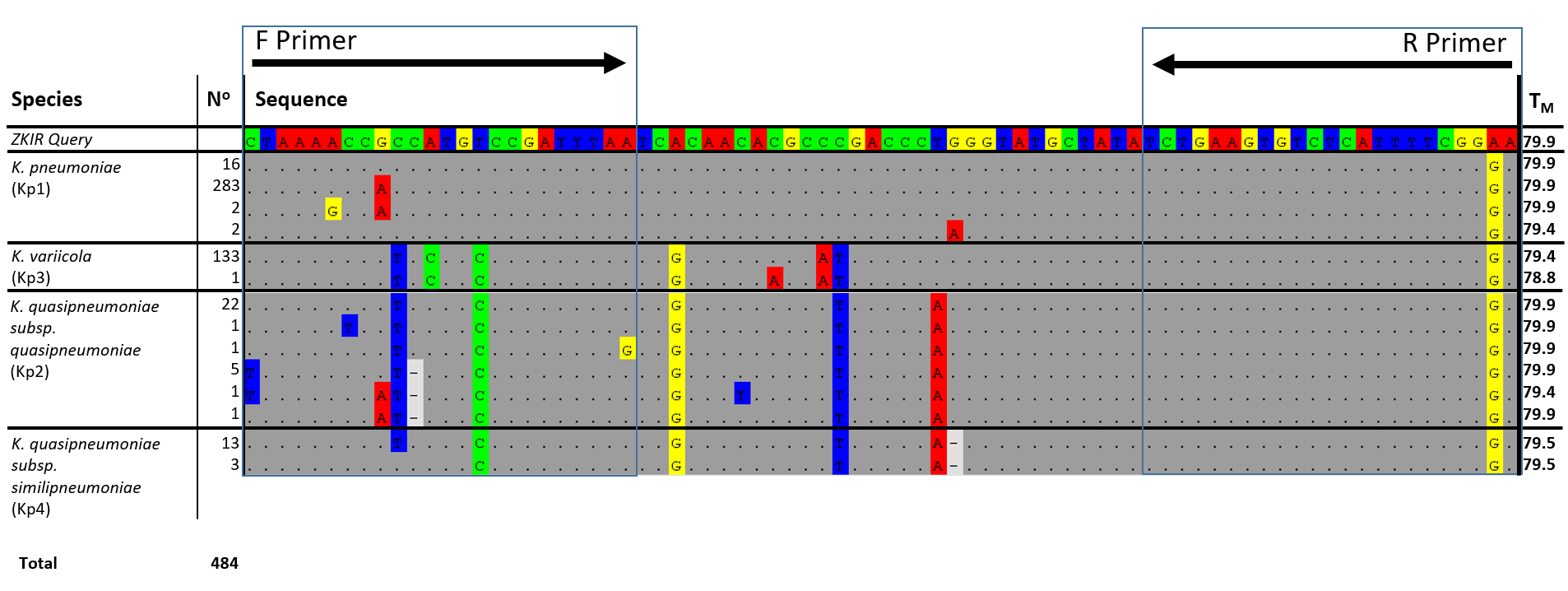

Supplement: Supplemental Material [file KGMI_A_2118500_SM9138.zip › Supplementary Figure 1.tiff]

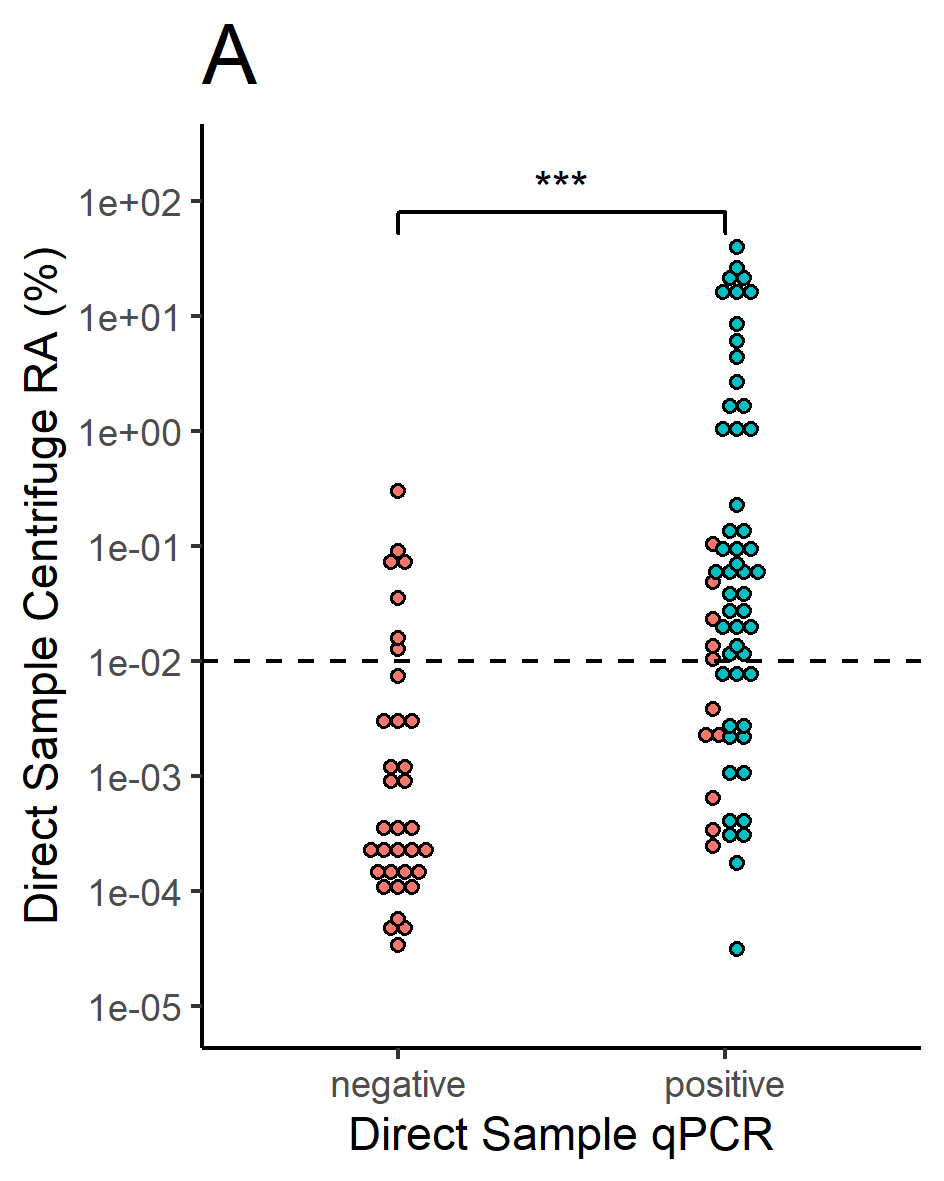

Supplement: Supplemental Material [file KGMI_A_2118500_SM9138.zip › Supplementary Figure 2A.tiff]

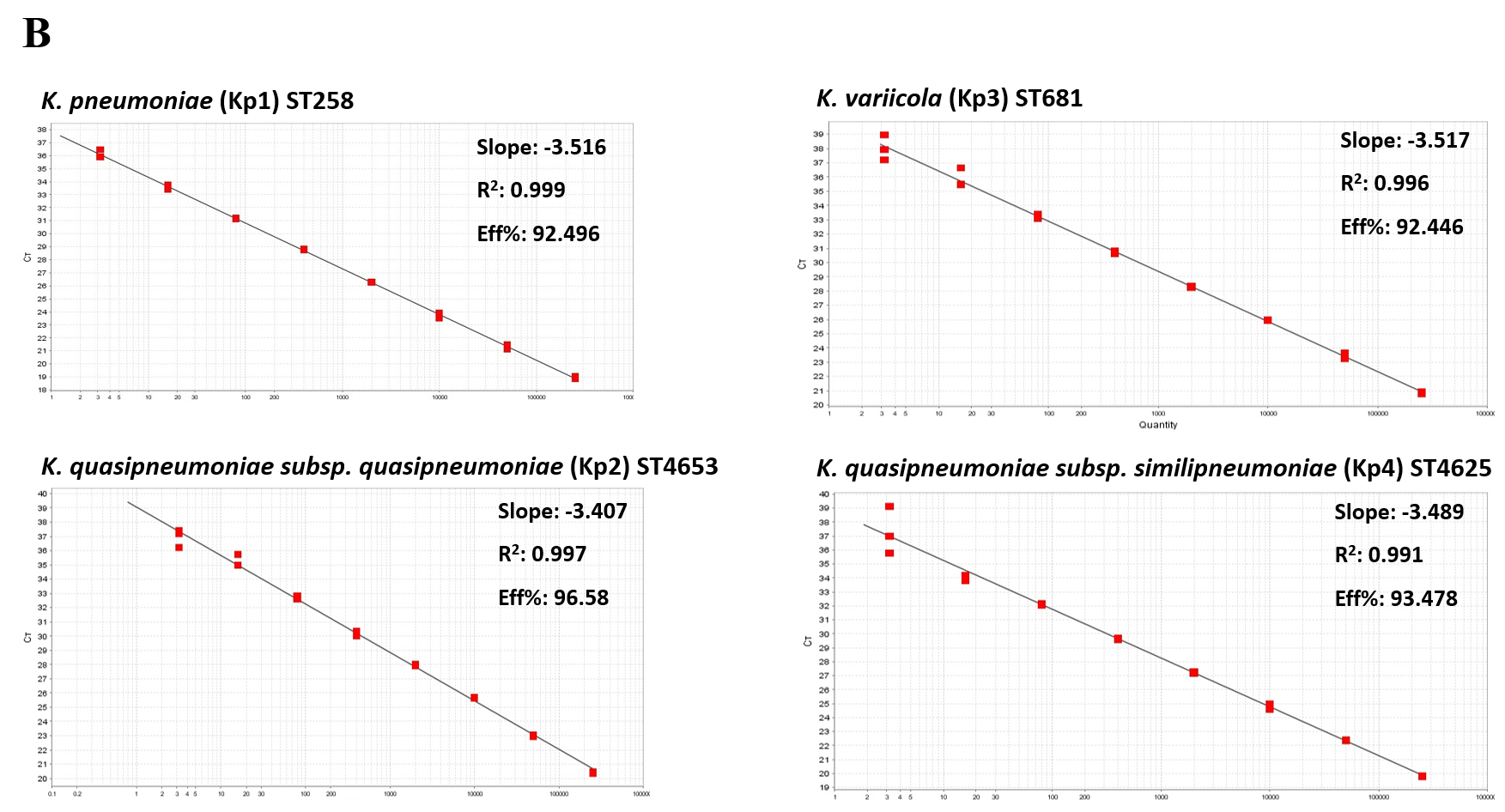

Supplement: Supplemental Material [file KGMI_A_2118500_SM9138.zip › Supplementary Figure 2B.tiff]

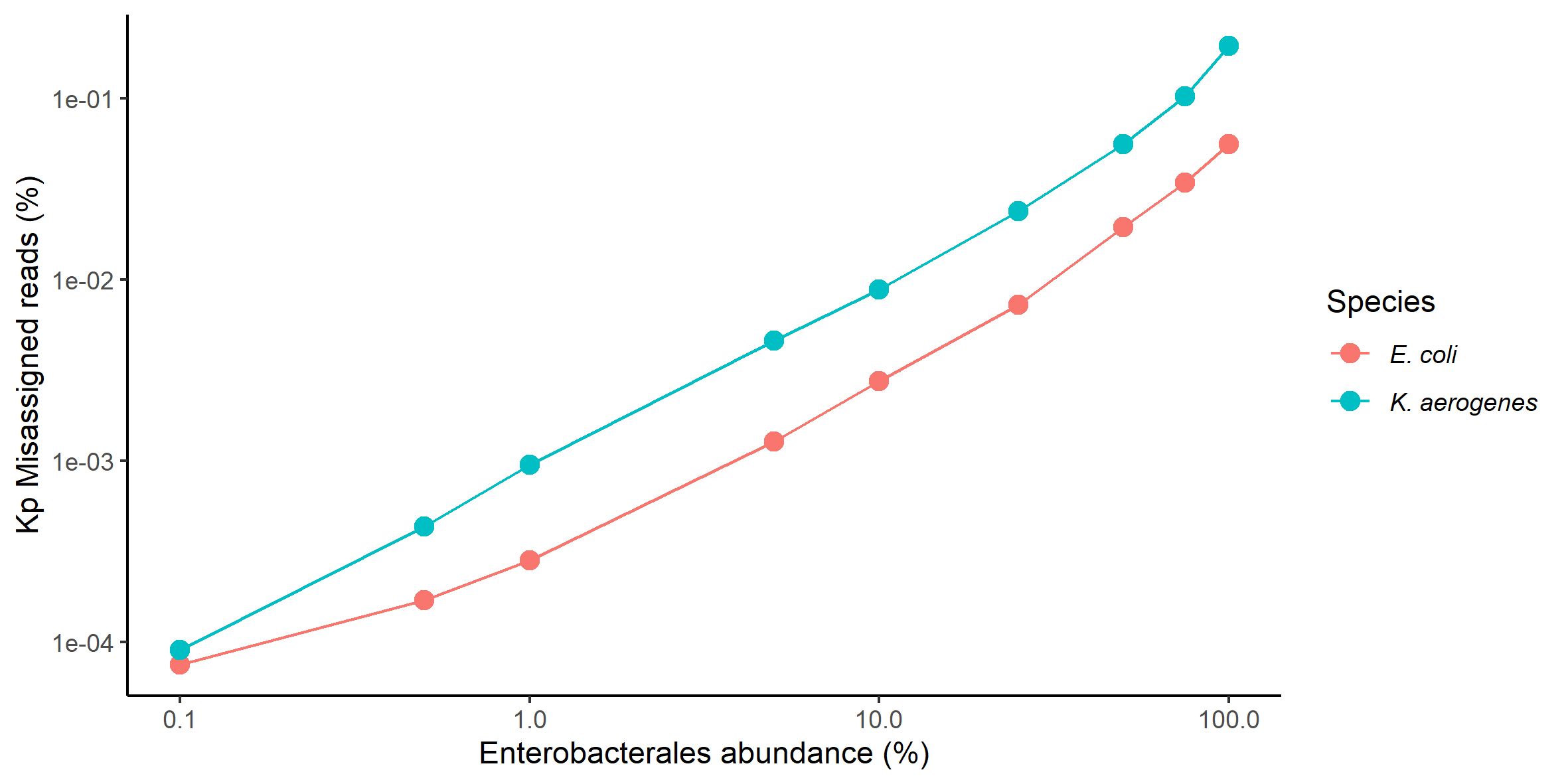

Supplement: Supplemental Material [file KGMI_A_2118500_SM9138.zip › Supplementary Figure 3.tiff]
